# International students – support and integration initiatives at Medical Faculties in Germany

**DOI:** 10.3205/zma001208

**Published:** 2018-11-30

**Authors:** Daniel Huhn, Christoph Nikendei

**Affiliations:** 1 University of Heidelberg, University Hospital for General Internal and Psychosomatic Medicine, Centre for Psychosocial Medicine, Heidelberg, Germany

## Editorial

Did you know that international students make up more than 12% of students at German medical faculties? In absolute numbers, this means that almost 12,000 international students are currently enrolled in German medical schools. Please note that the term ‘international’ does not mean young people living in Germany with a migrant background in their (family) biography, but exclusively refers to people coming to Germany with the intention of pursuing their university studies. So why do so many young people leave their home country and culture to study in Germany? The quality of medical education and the comparably low university fees are key incentives for many young academics.

Although there are a large number of international students at German medical faculties, many questions concerning this interesting group remain unanswered. Also, comparatively little work has been published in German-speaking countries regarding international students. However, existing research shows that international medical students report more stress symptoms, achieve poorer results in written, oral, and practical exams and show extended study duration as well as higher dropout rates. Particularly, the first semesters can be regarded as a critical phase in which international students are confronted with language deficits and cultural obstacles.

An improved understanding of international students within the framework of German medical education can have advantages for both sides: if their needs and their concerns become clearer, greater attention can be paid to them – providing these students with a greater chance of a successful academic career. Simultaneously, these international students are a valuable chance to gain a deeper understanding of the heterogeneity and complexity of people and cultures. Mutual learning with and from each other offers many opportunities for all those involved – it would be a pity to let these pass by.

We are, therefore, pleased to have the opportunity of presenting this "International Medical Students" issue to our reader and hope to provide this particular group of students with a forum within which one or two open questions can be answered. As editors, we hope that the commentary presented in this context as well as the four research papers and four project reports will be received with great interest. We hope that this issue raises awareness for the special situation of international medical students and offers practical suggestions for the implementation of initiatives at other faculties. Perhaps this issue will even initiate a much welcomed progressive scientific discussion of this hitherto underrepresented field of research.

In this issue you will first find a general commentary on this topic, followed by contributions regarding students' first steps in their studies as well as networking among international students. Subsequently, two Peer-assisted Learning (PAL) programs to support international students are introduced as well as a further support offer for students in clinical semesters. A further contribution deals with the question of the extent to which tertials spent abroad during the Practical Year affect the performance of German students. Finally, the "Symposium International Medical Students", launched in 2017, will be featured.

Each contribution was evaluated by three reviewers; two of them were selected in the conventional way in the course of the peer review process, one of them was himself an international student. We felt it was important to ensure that the contributions written *about* international students were also reviewed *from the perspective of* an international student.

Timo Astfalk's and colleagues' commentary "On center field or at the sidelines?" raises the question of to what extent a deficit-oriented perception of international medical students and related specific support offers are helpful in the first place for this group and if stronger integration into existing student bodies would yield greater benefits [1].

With regard to the two contributions to the study start, Yassin Karay and colleagues present the program "Studienstart International" of the University of Cologne [2], and Wendelin Marmon and colleagues present their program " Welcome, Orientation, Language Training" of the Charité in Berlin [3]. Both programs are designed to make it easier for international students to start their academic studies, and were evaluated in terms of participant satisfaction.

In the form of a qualitative analysis, Timo Astfalk and Brigitte Müller-Hilke approach the topic of networking among international students in pre-clinical semesters and make comparisons with networks of German students [4].

In a separate article ("Interactive student-led examination preparation course"), we present a longitudinal tutorial program that prepares international medical students in Heidelberg for examinations in the pre-clinical semesters [5]. In "Peer-teaching International" Daisy Rotzoll and colleagues also present a PAL project which is offered in Leipzig for Erasmus students arriving there in order to better prepare them for their one-year stay in Germany [6].

Furthermore, Holger Lenz and his colleagues present the support program – "An equal opportunity for everyone?!" – for international students in clinical semesters at the LMU in Munich, which focuses on getting to know and practicing communication strategies [7].

In their research, Sylvère Störmann and Matthias Angstwurm ("What do international health electives and state examination scores have in common?") do not look at international students in Germany, but analyse German ‘international’ students studying abroad for parts of their practical year, and try to find out whether this experience has a positive impact on the academic achievements [8].

Finally, Henrike Schulze and colleagues have summarized the "Symposium International Medical Students" of the German Medical Students Association, which took place for the first time in May 2017 in Hanover [9].

## Thanks

We would like to thank the editors of the GMS Journal for Medical Education, Prof. Dr. med. Martin Fischer and Dr. med. Götz Fabry, for their support and giving us the opportunity of realising this exciting issue. We thank all authors for their enthusiastic cooperation and for their valuable contributions as well as all reviewers for their untiring and consistent support of the review process. Our heartfelt thanks go to Beate Hespelein (editorial office of the GMS Journal for Medical Education) for her competent administration of the submitted contributions and coordination of the peer review process as well as for her unwavering capacity of never losing sight of the bigger picture.

## Competing interests

The authors declare that they have no competing interests. 
